# Treatment of pure aortic regurgitation using surgical or transcatheter aortic valve replacement between 2018 and 2020 in Germany

**DOI:** 10.3389/fcvm.2023.1091983

**Published:** 2023-05-02

**Authors:** Vera Oettinger, Ingo Hilgendorf, Dennis Wolf, Peter Stachon, Adrian Heidenreich, Manfred Zehender, Dirk Westermann, Klaus Kaier, Constantin von zur Mühlen

**Affiliations:** ^1^ Department of Cardiology and Angiology, University Heart Center, Medical Center—University of Freiburg, Faculty of Medicine, University of Freiburg, Freiburg, Germany; ^2^ Center for Big Data Analysis in Cardiology (CeBAC), Department of Cardiology and Angiology, University Heart Center, Medical Center—University of Freiburg, Faculty of Medicine, University of Freiburg, Freiburg, Germany; ^3^ Institute of Medical Biometry and Statistics, Faculty of Medicine and Medical Center—University of Freiburg, Freiburg, Germany

**Keywords:** aortic regurgitation, transcatheter aortic valve replacement, transcatheter aortic valve implantation, surgical aortic valve replacement, in-hospital outcomes, national electronic health records

## Abstract

**Background:**

In pure aortic regurgitation, transcatheter aortic valve replacement (TAVR) is not yet used on a regular base. Due to constant development of TAVR, it is necessary to analyze current data.

**Methods:**

By use of health records, we analyzed all isolated TAVR or surgical aortic valve replacements (SAVR) for pure aortic regurgitation between 2018 and 2020 in Germany.

**Results:**

4,861 procedures—4,025 SAVR and 836 TAVR—for aortic regurgitation were identified. Patients treated with TAVR were older, showed a higher logistic EuroSCORE, and had more pre-existing diseases. While results indicate a slightly higher unadjusted in-hospital mortality for transapical TAVR (6.00%) vs. SAVR (5.71%), transfemoral TAVR showed better outcomes, with self-expanding compared to balloon-expandable transfemoral TAVR having significantly lower in-hospital mortality (2.41% vs. 5.17%; *p* = 0.039). After risk adjustment, balloon-expandable as well as self-expanding transfemoral TAVR were associated with a significantly lower mortality vs. SAVR (balloon-expandable: risk adjusted OR = 0.50 [95% CI 0.27; 0.94], *p* = 0.031; self-expanding: OR = 0.20 [0.10; 0.41], *p* < 0.001). Furthermore, the observed in-hospital outcomes of stroke, major bleeding, delirium, and mechanical ventilation >48 h were significantly in favor of TAVR. In addition, TAVR showed a significantly shorter length of hospital stay compared to SAVR (transapical: risk adjusted Coefficient = −4.75d [−7.05d; −2.46d], *p* < 0.001; balloon-expandable: Coefficient = −6.88d [−9.06d; −4.69d], *p* < 0.001; self-expanding: Coefficient = −7.22 [−8.95; −5.49], *p* < 0.001).

**Conclusions:**

TAVR is a viable alternative to SAVR in the treatment of pure aortic regurgitation for selected patients, showing overall low in-hospital mortality and complication rates, especially with regard to self-expanding transfemoral TAVR.

## Introduction

Transcatheter aortic valve replacement (TAVR) has shown rapid developments (1–3). Its use was initially limited to patients with aortic valve stenosis (4–6). For this indication, TAVR is now a common therapy in the United States (7) and Germany (2, 6). However, in pure aortic regurgitation, TAVR is not yet used on a regular base. According to the current European (8) and American (9) guidelines for the management of valvular heart disease, surgery is the standard when valve replacement is required for aortic regurgitation; TAVR might be taken into account in selected patients with an aortic regurgitation who are not eligible for surgical aortic valve replacement (SAVR).

We previously analyzed TAVR in aortic regurgitation in Germany from 2008 to 2015, and concluded that TAVR may be a safe option for treating aortic regurgitation (5). However, in the early years of TAVR, its use in the context of aortic regurgitation was even rarer than it is today. In addition, due to the constant development of TAVR, it is necessary to analyze current data to gain further insights.

We have now compared all patients who were treated with SAVR or TAVR for pure aortic regurgitation between 2018 and 2020 in Germany. Our analysis thus represents the current state of research in this area. Furthermore, we distinguish between the different access routes of TAVR (i.e., transfemoral (TF) or transapical (TA)), and valve types (balloon-expandable (BE) or self-expanding (SE)).

## Material and methods

Since 2005, the data of all hospital stays in Germany can be used for scientific purposes via Diagnosis Related Groups (DRG) statistics, which are collected by the Research Data Center of the Federal Bureau of Statistics (DESTATIS). These data on hospital stays, including diagnoses and procedures, are a valuable nationwide data source on in-hospital patient treatment and represent a virtually complete collection of all hospital stays in German centers that are reimbursed in accordance with the DRG system. From this database, data on all isolated SAVR and TAVR procedures conducted between 2018 and 2020 were extracted (2, 10, 11). We defined the isolated procedures using OPS codes, including all aortic valve procedures and excluding concomitant procedures at the mitral valve, tricuspid valve procedures, coronary artery bypass graft procedures and Maze procedures. We included data on patients with pure aortic regurgitation only (main or secondary diagnosis: I35.1, I35.8, I35.9, I06.1, I06.8 or I06.9). Thus, patients with a documented aortic valve stenosis (main or secondary diagnosis: I35.0, I35.2, I06.0, I06.2) were excluded. Since our focus was on isolated SAVR and TAVR procedures, we also excluded those patients with a concomitant cardiac surgery or a percutaneous coronary intervention (5).

Furthermore, we used a set of baseline characteristics to describe the underlying diseases and the risk factors of the patients studied. ICD codes have been previously discussed in more detail (2). Using the European System for Cardiac Operative Risk Evaluation (EuroSCORE) (12), a “best-case scenario” risk score was estimated. In addition to age and sex, we utilized the ICD codes for a chronic pulmonary disease (J43*, J44*), a neurological dysfunction (I69*, G81*, R48*), a previous cardiac surgery (Z95.1–Z95.4), a serum creatinine >200 µmol/L (N18.0, N18.84, N18.5), an active endocarditis (I33*), unstable angina (I20.0), a recent myocardial infarction (I25.20), and a pulmonary hypertension (I27*). An inconspicuous state was supposed for the “preoperative state” and “left ventricular function” due to the lack of data (the “best-case”, i.e., no emergency, preserved left ventricular function). To allow comparison of baseline risk factors in patients treated with transcatheter or surgical aortic valve replacement, the logistic EuroSCORE was evaluated assuming isolated aortic valve replacement.

In-hospital outcomes are in-hospital mortality, major bleeding with more than five units of red blood cells needed during the in-hospital stay (OPS: 8-800.c1 et seqq.), stroke (ICD: I63* and I64), and postoperative delirium (ICD: F05). Furthermore, health economic outcomes comprise the length of hospital stay, reimbursement, and mechanical ventilation >48 h, which are provided by DESTATIS' own variable pool.

Due to the lack of codes indicating missing data, an attribution of missing values could not be performed. If a clinical characteristic was not included in the patient's electronic health record, it was presumed not to be present.

To calculate differences in outcomes between groups, Student's t-test and chi-square test were applied for continuous and categorical variables, respectively. In addition, we used multivariable logistic or linear regression models and included 21 baseline characteristics as potential confounders, as listed in Table 1. We included a random intercept at the hospital level to account for the correlation of error terms of patients treated at the same hospital. Based on these confounder-adjusted regression analyzes, predicted rates and means were calculated using marginal standardization (13). The results of the regression analyzes are presented in the Supplementary Appendix S1.

**Table 1:** Baseline characteristics of patients with pure aortic regurgitation between 2018 and 2020 in Germany.

	SAVR		TA-TAVR		TF-TAVR BE		TF-TAVR SE	
N	4,025		50		329		457	
2018	34.51%		34.00%		30.70%		32.82%	
2019	33.76%		26.00%		32.52%		31.95%	
2020	31.73%		40.00%		36.78%		35.23%	
Female	25.42%		38.00%		25.84%		40.26%	
Age in years, mean / SD	62.75	13.58	76.00	9.15	76.27	9.54	77.25	8.11
Logistic EuroSCORE, mean / SD	4.93	5.69	19.08	14.68	18.23	13.45	17.66	12.54
NYHA II	15.65%		xxx		14.59%		xxx	
NYHA III or IV	33.81%		56.00%		52.58%		56.02%	
CAD	14.61%		56.00%		49.24%		42.89%	
Arterial hypertension	58.04%		70.00%		69.30%		67.40%	
Previous MI within 4 months	0.57%		0.00%		0.91%		1.09%	
Previous MI within 1 year	0.52%		xxx		1.22%		xxx	
Previous MI after 1 year	1.57%		xxx		5.47%		xxx	
Previous CABG	1.52%		24.00%		25.53%		20.13%	
Previous cardiac surgery	7.68%		68.00%		71.12%		62.80%	
Peripheral vascular disease	2.83%		24.00%		6.69%		7.66%	
Carotid disease	2.24%		14.00%		4.56%		3.50%	
COPD	7.16%		16.00%		9.73%		11.82%	
Pulmonary hypertension	8.62%		18.00%		24.01%		25.60%	
Renal disease, GFR <15 ml/min	1.71%		xxx		xxx		2.19%	
Renal disease, GFR <30 ml/min	1.81%		xxx		xxx		7.00%	
Atrial fibrillation	44.47%		40.00%		44.07%		50.11%	
Diabetes mellitus	12.20%		18.00%		18.24%		19.04%	
Emergency	14.04%		12.00%		19.15%		17.07%	

BE, balloon-expandable; CABG, coronary artery bypass graft; CAD, coronary artery disease; COPD, chronic obstructive pulmonary disease; EuroSCORE, European System for Cardiac Operative Risk Evaluation; GFR, glomerular filtration rate; MI, myocardial infarction; N, number of procedures; NYHA, New York Heart Association; SAVR, surgical aortic valve replacement; SD, standard deviation; SE, self-expanding; TA, transapical; TAVR, transcatheter aortic valve replacement; TF, transfemoral.

xxx: The Research Data Center of the Federal Bureau of Statistics censored all values that could allow conclusions to be drawn about a single patient or a specific hospital.

Using the logistic EuroSCORE, a “best-case scenario” risk score was estimated. An inconspicuous state was supposed for the “preoperative state” and “left ventricular function” due to the lack of data (the “best-case”, i.e. no emergency, preserved left ventricular function).

No adjustment for multiple testing took place. Therefore, the *p*-values may not be interpreted as confirmatory but as descriptive. All analyses were carried out using Stata 17 (Stata Corp, College Station, Texas).

## Results

### Baseline characteristics

**Figure 1:**
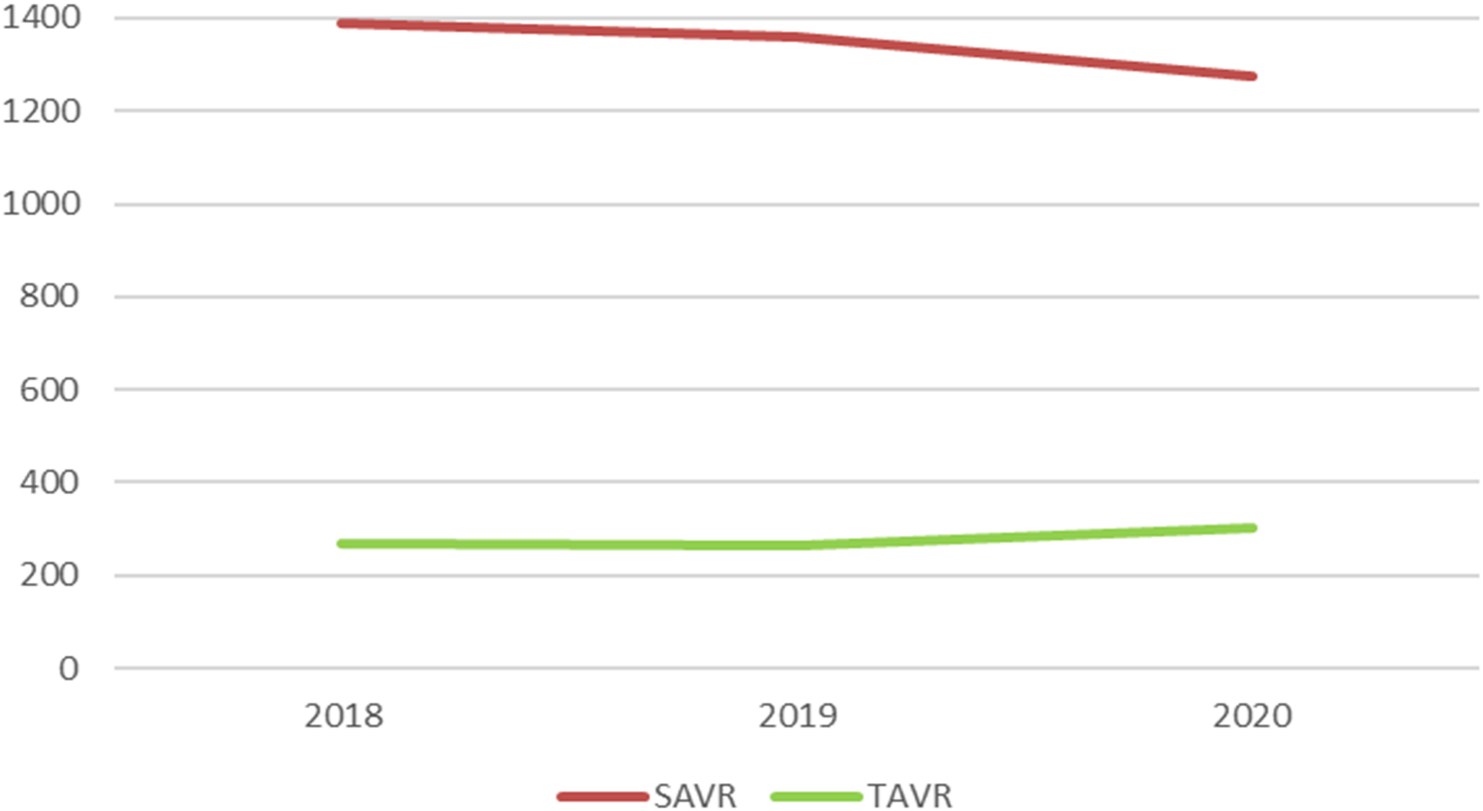
Numbers of SAVR and TAVR for pure aortic regurgitation between 2018 and 2020 in Germany. SAVR, surgical aortic valve replacement; TAVR, transcatheter aortic valve replacement.

Between 2018 and 2020, a total of 4,861 patients were treated for a pure aortic regurgitation with either TAVR or SAVR (Table 1). Of these, 4,025 received SAVR, 50 TA-TAVR, 329 BE TF-TAVR, and 457 SE TF-TAVR. While the number of SAVR procedures decreased from 1,389 to 1,277 between 2018 and 2020, the number of TAVR increased from 268 to 302 (Figure 1).

Age was noticeably higher in TAVR than SAVR (SAVR vs. TA vs. BE vs. SE: 62.75a, 76.00a, 76.27a, 77.25a). The same applied to the logistic EuroSCORE, which was higher in TAVR (4.93, 19.08, 18.23, 17.66). Likewise, patients in the TAVR groups had more pre-existing diseases, e.g., more higher grade heart failure NYHA III/IV (33.81%, 56.00%, 52.58%, 56.02%), coronary artery disease (14.61%, 56.00%, 49.24%, 42.89%), previous coronary artery bypass graft (CABG; 1.52%, 24.00%, 25.53%, 20.13%) or previous cardiac surgery (7.68%, 68.00%, 71.12%, 62.80%). The rate of peripheral vascular disease was highest in TA-TAVR (2.83%, 24.00%, 6.69%, 7.66%).

### Unadjusted in-hospital outcomes of patients treated for aortic regurgitation

When comparing the unadjusted in-hospital mortality between SAVR and the analyzed different access routes of TAVR, results indicate a slightly higher mortality rate for TA-TAVR with 6.00% vs. SAVR with 5.71% (Table 2). However, TF-TAVR shows better outcomes than SAVR, with lowest rate of in-hospital mortality in self-expanding TF-TAVR. In addition, self-expanding TF-TAVR is associated with a significantly lower mortality rate in a direct comparison to balloon-expandable TF-TAVR (BE 5.17%, SE 2.41%; *p* = 0.039).

**Table 2:** Unadjusted in-hospital outcomes of patients treated for aortic regurgitation between 2018 and 2020.

	SAVR		TA-TAVR		TF-TAVR BE		TF-TAVR SE		*p*-value
									BE vs SE
									TF-TAVR
N	4,025		50		329		457		
In-hospital mortality	5.71%		6.00%		5.17%		2.41%		0.039
Stroke	5.66%		0.00%		2.43%		2.19%		0.822
Major bleeding >5 units	21.64%		8.00%		3.04%		2.19%		0.455
Delirium	15.93%		12.00%		6.38%		6.13%		0.884
Mechanical ventilation >48 h	17.61%		6.00%		4.26%		2.84%		0.284
Length of hospital stay (mean, SD)	17.80d	14.91d	15.82d	10.35d	13.75d	9.94d	13.69d	9.75d	0.933
Reimbursement (mean, SD)	24,906€	20,037€	31,005€	7,181€	27,777€	7,289€	27,213€	5,389€	0.213

BE, balloon-expandable; N, number of procedures; SAVR, surgical aortic valve replacement; SD, standard deviation; SE, self-expanding; TA, transapical; TAVR, transcatheter aortic valve replacement; TF, transfemoral.

*p*-values based on chi-square test or t-test as appropriate.

**Figure 2:**
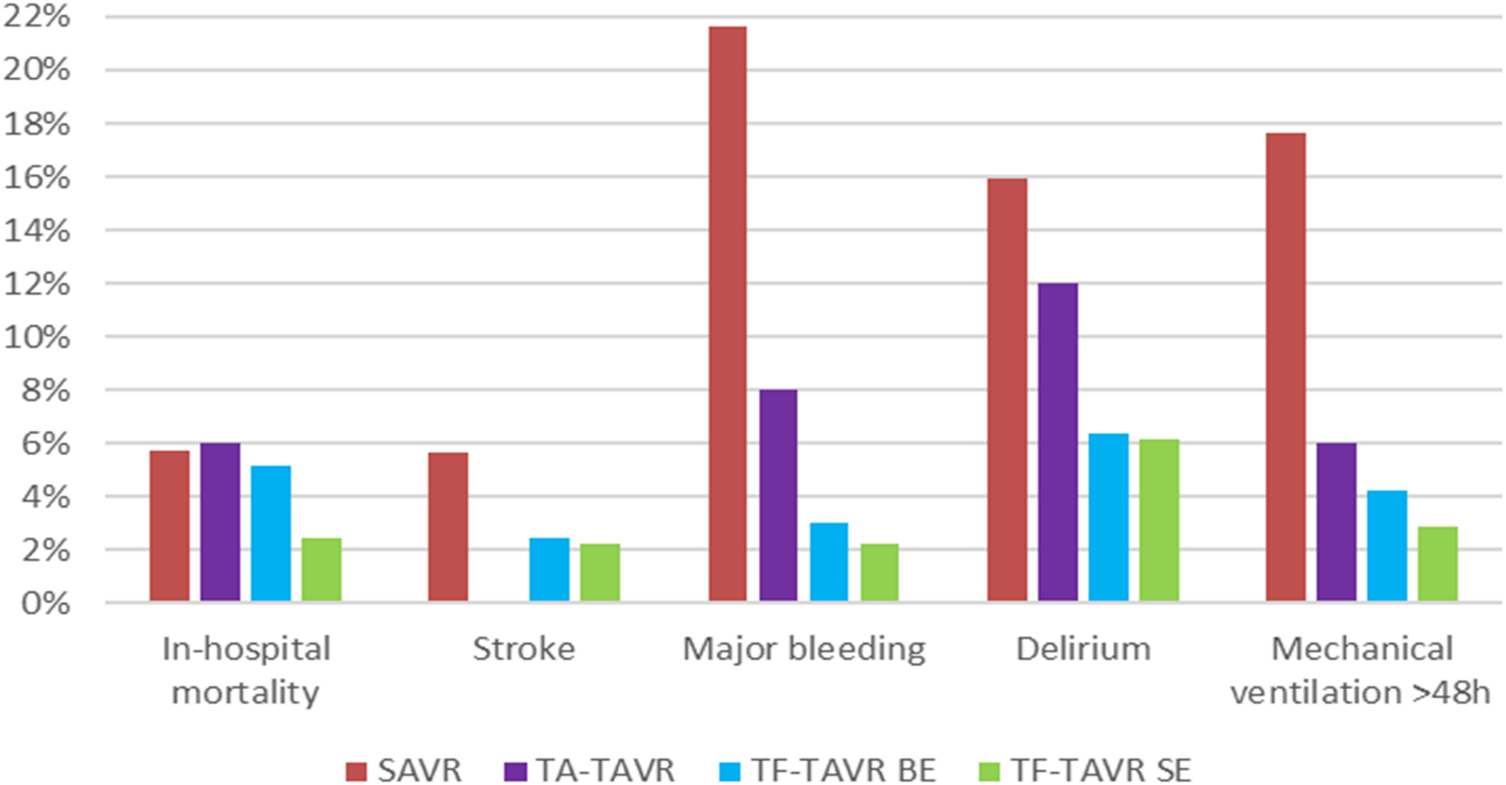
Unadjusted in-hospital outcomes of patients treated for aortic regurgitation between 2018 and 2020 in Germany. BE, balloon-expandable; SAVR, surgical aortic valve replacement; SE, self-expanding; TA, transapical; TAVR, transcatheter aortic valve replacement; TF, transfemoral.

Regarding the unadjusted in-hospital outcomes of stroke, major bleeding, delirium, and mechanical ventilation >48 h, results are in favor of TAVR (Figure 2). Rates of stroke, major bleeding, delirium, and mechanical ventilation >48 h did not differ significantly between balloon-expandable and self-expanding TF-TAVR. Complication rates of TA-TAVR are higher than TF-TAVR for major bleeding, delirium, and mechanical ventilation >48 h. Only the rate of stroke was 0.00% in TA-TAVR, which should be seen in the context of the small number of only 50 patients in TA-TAVR.

TAVR also showed a shorter length of hospital stay, with lowest rates in TF-TAVR (SAVR 17.80d; TA 15.82d; BE 13.75d; SE 13.69d). Reimbursement was highest in TA-TAVR at 31,005€ and lowest in SAVR at 24,906€.

### Risk-adjusted in-hospital outcomes of patients treated for aortic regurgitation

**Figure 3:**
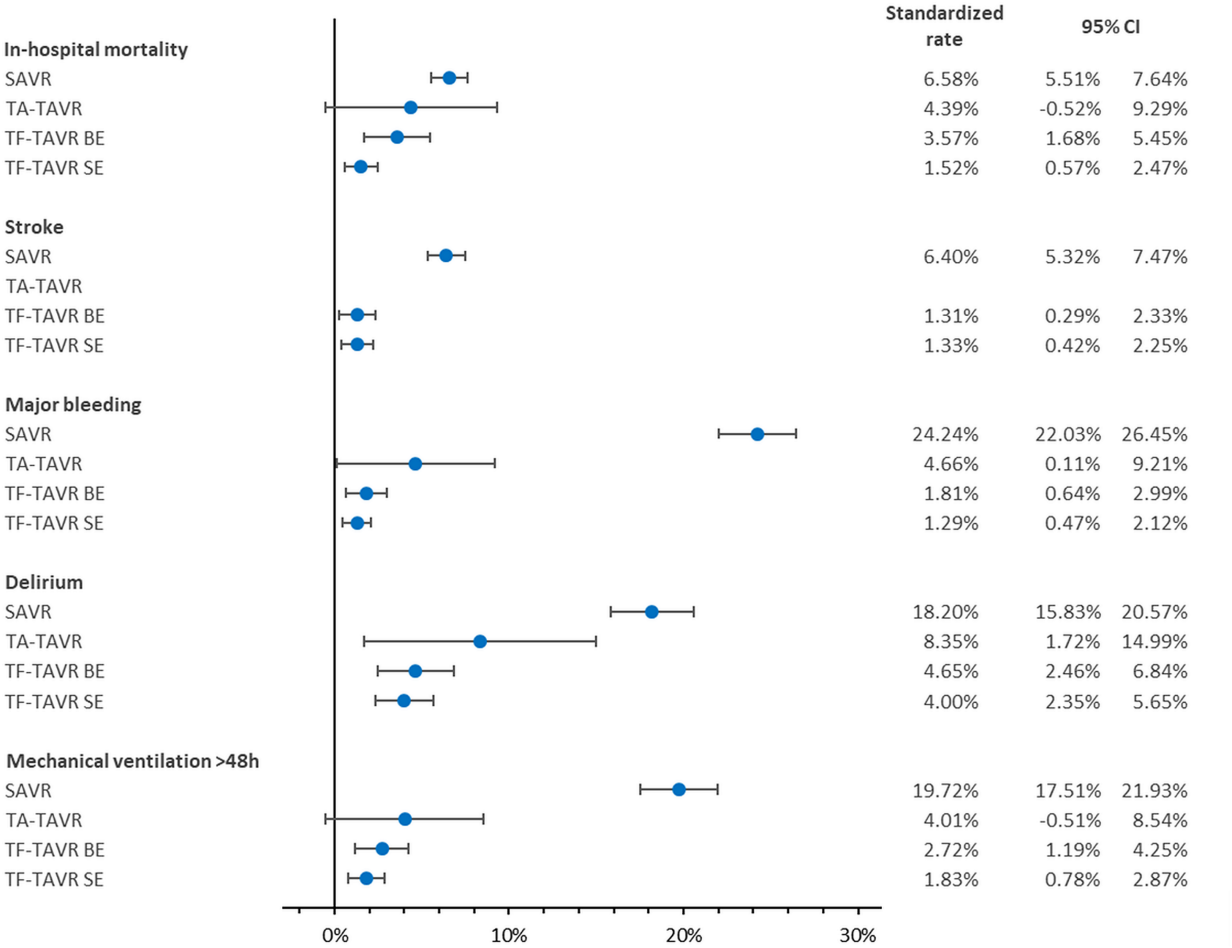
Standardized rates of in-hospital outcomes of patients treated for aortic regurgitation between 2018 and 2020 in Germany. BE, balloon-expandable; CI, confidence interval; SAVR, surgical aortic valve replacement; SE, self-expanding; TA, transapical; TAVR, transcatheter aortic valve replacement; TF, transfemoral. Values of stroke in TA-TAVR could not be calculated due to a stroke rate of 0.00% in TA-TAVR.

After risk adjustment, balloon-expandable as well as self-expanding TF-TAVR were associated with a significantly lower mortality rate vs. SAVR as reference (TA: risk adjusted OR = 0.63 [95% CI 0.18; 2.23], *p* = 0.476; BE: OR = 0.50 [0.27; 0.94], *p* = 0.031; SE: OR = 0.20 [0.10; 0.41], *p* < 0.001; Table 3). Accordingly, balloon-expandable as well as self-expanding TF-TAVR showed significantly better standardized rates of in-hospital mortality vs. SAVR (SAVR: 6.58% [5.51%; 7.64%]; TA: 4.39% [−0.52%; 9.29%]; BE: 3.57% [1.68%; 5.45%]; SE: 1.52% [0.57%; 2.47%]; Figure 3).

**Table 3:** Regression results of in-hospital outcomes of patients treated for aortic regurgitation between 2018 and 2020.

	Odds ratios/Coefficients				Standardized rates/mean		
	OR	*p*-value	95% CI		Standardized rate	95% CI	
**In-hospital mortality**							
SAVR	1 (reference)				6.58%	5.51%	7.64%
TA-TAVR	0.63	0.476	0.18	2.23	4.39%	−0.52%	9.29%
TF-TAVR BE	0.50	0.031	0.27	0.94	3.57%	1.68%	5.45%
TF-TAVR SE	0.20	0.000	0.10	0.41	1.52%	0.57%	2.47%
**Stroke**							
SAVR	1 (reference)				6.40%	5.32%	7.47%
TA-TAVR	xx	xx	xx	xx	xx	xx	xx
TF-TAVR BE	0.17	0.000	0.07	0.42	1.31%	0.29%	2.33%
TF-TAVR SE	0.17	0.000	0.08	0.39	1.33%	0.42%	2.25%
**Major bleeding**							
SAVR	1 (reference)				24.24%	22.03%	26.45%
TA-TAVR	0.12	0.000	0.04	0.37	4.66%	0.11%	9.21%
TF-TAVR BE	0.04	0.000	0.02	0.09	1.81%	0.64%	2.99%
TF-TAVR SE	0.03	0.000	0.02	0.06	1.29%	0.47%	2.12%
**Delirium**							
SAVR	1 (reference)				18.20%	15.83%	20.57%
TA-TAVR	0.37	0.039	0.14	0.95	8.35%	1.72%	14.99%
TF-TAVR BE	0.19	0.000	0.11	0.32	4.65%	2.46%	6.84%
TF-TAVR SE	0.16	0.000	0.10	0.25	4.00%	2.35%	5.65%
**Mechanical ventilation >48 h**							
SAVR	1 (reference)				19.72%	17.51%	21.93%
TA-TAVR	0.14	0.002	0.04	0.49	4.01%	−0.51%	8.54%
TF-TAVR BE	0.09	0.000	0.05	0.17	2.72%	1.19%	4.25%
TF-TAVR SE	0.06	0.000	0.03	0.11	1.83%	0.78%	2.87%
	Coefficient	*p*-value	95% CI		Standardized mean	95% CI	
**Length of hospital stay**							
SAVR	(reference)				18.94d	17.99d	19.90d
TA-TAVR	−4.75d	0.000	−7.05d	−2.46d	14.19d	11.92d	16.45d
TF-TAVR BE	−6.88d	0.000	−9.06d	−4.69d	12.06d	10.14d	13.99d
TF-TAVR SE	−7.22d	0.000	−8.95d	−5.49d	11.72d	10.17d	13.27d
**Reimbursement**							
SAVR	(reference)				25,841.58€	24,850.03€	26,833.14€
TA-TAVR	2,708.97€	0.038	152.25€	5,265.69€	28,550.55€	26,309.01€	30,792.10€
TF-TAVR BE	−799.40€	0.486	−3,049.58€	1,450.79€	25,042.19€	23,157.85€	26,926.53€
TF-TAVR SE	−1,666.56€	0.047	−3,307.30€	−25.82€	24,175.02€	22,964.89€	25,385.16€

BE, balloon-expandable; CI, confidence interval; N, number of procedures; OR, odds ratio; SAVR, surgical aortic valve replacement; SD, standard deviation; SE, self-expanding; TA, transapical; TAVR, transcatheter aortic valve replacement; TF, transfemoral.

xx: Values of stroke in TA-TAVR could not be calculated due to a stroke rate of 0.00% in TA-TAVR.

With regard to the risk-adjusted in-hospital outcomes of stroke, major bleeding, delirium, and mechanical ventilation >48 h, ORs were continuously in favor of TAVR. Values for stroke in TA-TAVR could not be calculated because no stroke was reported in any of the 50 patients. Besides that, the best results again were seen in TF-TAVR for major bleeding, delirium, and mechanical ventilation >48 h. The same was reflected in the corresponding standardized rates.

In relation to resource utilization parameters, TAVR showed a significantly shorter length of hospital stay compared to SAVR and the length of stay was shortest in SE TF-TAVR (TA: risk adjusted Coefficient = −4.75d [−7.05d; −2.46d], *p* < 0.001; BE: Coefficient = −6.88d [−9.06d; −4.69d], *p* < 0.001; SE: Coefficient = −7.22 [−8.95; −5.49], *p* < 0.001). This was also reflected in the standardized means (SAVR: 18.94d [17.99d; 19.90d]; TA: 14.19d [11.92d; 16.45d]; BE: 12.06d [10.14d; 13.99d]; SE: 11.72d [10.17d; 13.27d]). For reimbursement, the results were mixed: It was significantly higher in TA-TAVR than SAVR [Coefficient = 2,708.97€ (152.25€; 5,265.69€), *p* = 0.038] and significantly lower in SE TF-TAVR [Coefficient = −1,666.56€ (−3,307.30€; −25.82€), *p* = 0.047].

## Discussion

In this study, we examined 4,861 SAVR and TAVR for pure aortic regurgitation between 2018 and 2020 in Germany. Despite a higher age and logistic EuroSCORE as well as overall more pre-existing diseases in patients with TAVR compared to SAVR, we observed convincing results of TAVR in the analyzed patient collective. Especially self-expanding TF-TAVR achieves a significantly lower in-hospital mortality as well as noticeably lower complication rates.

For aortic valve stenosis, TAVR is a common therapy in the United States (7) and Germany (2, 6). However, TAVR is not yet routinely used in pure aortic regurgitation, where a surgical approach is the standard when valve replacement is required. It should be noted that TAVR was used off-label in the treatment of aortic regurgitation in Germany between 2018 and 2020. According to the current European (8) and American (9) guidelines for the management of valvular heart disease, TAVR might be taken into account in selected patients with an aortic regurgitation who are not eligible for SAVR. However, with reference to the recommendations for aortic valve stenosis, TAVR is not recommended for patients with, for example, endocarditis or unsuitable anatomical conditions such as unfavorable aortic annular dimensions or a significant dilatation of the aortic root respectively ascending aorta (8). Another difficulty is a potentially insufficient amount of calcification, which may make TAVR more challenging (5).

We observe that the number of TAVR cases in aortic regurgitation is still small compared to SAVR. Nevertheless, compared to a previous analysis of TAVR in aortic regurgitation in Germany from 2008 to 2015 (5), we see a further growth of TAVR procedures. The increased TAVR numbers in 2020 are particularly surprising: Due to the COVID-19 pandemic with lockdown restrictions in 2020 in Germany, including postponing elective procedures to provide hospital resources for COVID-19 patients (14), lower numbers could have been expected, as this also was observed in acute ST-elevation myocardial infarction (15).

Furthermore, in contrast to a previously stated rise of TA-TAVR procedures (5), we now observe noticeably fewer cases compared to the steadily increasing TF-TAVR numbers. The same trend could be seen in TAVR for aortic valve stenosis in Germany some years ago (5). Several analyses saw favorable outcomes for TF-TAVR vs. TA-TAVR (16–18) in aortic valve stenosis and most centers prefer a transfemoral access (19). This parallels our results in aortic regurgitation. Also the European (8) and American (9) guidelines recommend TF-TAVR in aortic valve stenosis. Alternative access routes such as TA-TAVR are generally only performed if transfemoral is not possible (20, 21). This also explains the highest rate of peripheral vascular disease in TA-TAVR in our analysis when TF-TAVR cannot be used e.g., due to calcification.

Furthermore, we see better results especially with TF-TAVR vs. SAVR in aortic regurgitation, despite a higher age and logistic EuroSCORE as well as overall more pre-existing diseases in TAVR. Thus, TAVR has continued to be used mainly in patients suffering from aortic regurgitation with a comparatively high surgical risk in recent years, but has shown good results even in these patients.

A reason for the still high mortality of SAVR in aortic regurgitation could be its use in acute aortic regurgitation due to endocarditis or aortic dissection (5, 8, 22, 23). Again, it is important to mention that TAVR is not recommended for patients with, for example, endocarditis (8). Therefore, it must be taken into account that the observed patient groups are presumably preselected to a certain extent and may not be fully comparable.

Previously (5), we reported a markedly varying in-hospital mortality for TF-TAVR in aortic regurgitation between 2008 and 2015 (15.2% in 2011, 2.8% in 2015) as well as TA-TAVR (17.7% in 2012, 0% in 2014), which could be due to the low TAVR numbers in aortic regurgitation. Compared to the mean in-hospital mortality of 8.61% in TF-TAVR and 7.66% in TA-TAVR in that study, we now see a further decrease in in-hospital mortality. Regarding TF-TAVR in particular, the same applied for most in-hospital complications as well as length of hospital stay.

Arora et al. (24) as well as Isogai et al. (25) analyzed TAVR for aortic regurgitation in the United States in 2016-2017. They observed an in-hospital or 30-day mortality rate of between 2.4 and 3.3%, which is lower than our results for balloon-expandable TF-TAVR but consistent with or slightly higher than those for self-expanding TF-TAVR. The stroke rate was between 0.6 and 1.8%. Furthermore, Arora et al. (24) report a rate of major bleeding requiring blood transfusion of 2.2% in-hospital and 7.7% at 30 days, while Isogai et al. (25) saw bleeding complications in 17.4% with a blood transfusion rate of 8.0%. Mean length of hospital stay was between 3 and 4 days, which is noticeably shorter than ours. In addition, Isogai et al. (25) found that TAVR for aortic regurgitation vs. aortic valve stenosis was significantly associated with a higher risk of acute kidney injury (OR = 1.64, p < 0.001), cardiac tamponade (OR = 1.98, *p* = 0.0498), and prolonged hospital stay (OR = 1.59, *p* < 0.001), but not with in-hospital mortality (OR = 1.55, *p* = 0.058).

Comparing the current results with an analysis of ours on TF-TAVR for aortic valve stenosis in Germany in 2018 (26), the rates of in-hospital mortality and complications in aortic regurgitation have approached those of aortic valve stenosis. In addition, we observe advantages in favor of self-expanding vs. balloon-expandable TF-TAVR in aortic regurgitation. This is in contrast to findings in aortic valve stenosis, where broadly equivalent outcomes have been seen (26–28). The reasons for the advantages of self-expanding TF-TAVR in aortic regurgitation remain speculative. One hypothesis could be that our analysis might contain valve-in-valve TAVR with possibly better hemodynamic characteristics in SE TF-TAVR, resulting in less patient-prosthesis mismatch as well as lower transvalvular gradients after TAVR. This hypothesis may be of particular interest for long-term outcomes. However, the CENTER-study (29) also analyzed results of valve-in-valve TAVR in aortic valve stenosis and showed a lower rate of major bleeding after 30 days in SE valve-in-valve TAVR, but mortality did not differ significantly for in-hospital outcomes, after 30 days, and after one year.

Our analysis shows promising results, despite the off-label use of TAVR so far, and it is conceivable that TAVR will be used more frequently in aortic regurgitation in the future, even in selected patients with a lower surgical risk. This will require further research, particularly with new approved prostheses.

Our analysis has several strengths and limitations, as mentioned in previous studies (26, 30–34). First, a strength is the availability of complete national data of all TAVR and SAVR in pure aortic regurgitation. A limitation is the use of administrative data. Hence, coding errors can exist. Our model is missing some interesting parameters, for example information on the exact type of valve used in each procedure, the presence of a valve-in-valve procedure, the specific previous cardiac surgeries coded as well as echocardiographic parameters. The use of administrative data is limited in granularity. Furthermore, based on these codes in Germany, we used >5 units of red blood cells as definition of bleeding. This corresponds approximately to the bleeding classification Type 3 (life-threatening bleeding) according to the Valve Academic Research Consortium 3 (VARC-3) definition (35). In addition, since a long-term follow-up is not available due to the characteristics of our data source, we present an analysis of in-hospital outcomes.

## Conclusions

4,861 SAVR or TAVR for pure aortic regurgitation between 2018 and 2020 in Germany were examined. Taking into account the different selection criteria for TAVR or SAVR in aortic regurgitation, the data demonstrate that TAVR is a viable alternative to SAVR in the treatment of pure aortic regurgitation for selected patients, showing overall low in-hospital mortality and complication rates, especially with regard to self-expanding TF-TAVR.

## Data Availability

The raw data supporting the conclusions of this article will be made available by the authors, without undue reservation.
