# Supplementary material for: Treatment of pure aortic regurgitation using surgical or transcatheter aortic valve replacement between 2018 and 2020 in Germany
Source: Front Cardiovasc Med. 2023 May 2;10:1091983. doi: 10.3389/fcvm.2023.1091983 (PMC10187752; doi:10.3389/fcvm.2023.1091983)
Supplement: Supplementary file 1 [file Datasheet1.pdf]

Treatment of pure aortic regurgitation using surgical or transcatheter aortic valve replacement between 2018 and 2020 in Germany

Vera Oettinger, Ingo Hilgendorf, Dennis Wolf, Peter Stachon, Adrian Heidenreich, Manfred Zehender, Dirk Westermann, Klaus Kaier, Constantin von zur Mühlen

Regression results of patients with pure aortic regurgitation between 2018 and 2020 in Germany

	Mortality				Stroke				Major bleeding			
	OR	p	95% CI		OR	p	95% CI		OR	p	95% CI	
**SAVR**	1				1				1			
**TA-TAVR**	0.63	0.476	0.18	2.23	xx	xx	xx	xx	0.12	0.000	0.04	0.37
**TF-TAVR BE**	0.50	0.031	0.27	0.94	0.17	0.000	0.07	0.42	0.04	0.000	0.02	0.09
**TF-TAVR SE**	0.20	0.000	0.10	0.41	0.17	0.000	0.08	0.39	0.03	0.000	0.02	0.06
**Year**	0.87	0.092	0.74	1.02	1.00	0.956	0.84	1.17	1.01	0.816	0.92	1.12
**Women**	1.43	0.017	1.07	1.91	0.87	0.395	0.64	1.20	1.26	0.016	1.04	1.51
**Age**	1.01	0.039	1.00	1.03	0.98	0.011	0.97	1.00	1.01	0.016	1.00	1.02
**Logistic EuroSCORE**	1.00	0.835	0.97	1.02	1.11	0.000	1.08	1.14	1.00	0.759	0.98	1.02
**NYHA II**	0.53	0.015	0.31	0.88	0.71	0.115	0.47	1.08	0.58	0.000	0.44	0.75
**NYHA III or IV**	1.38	0.026	1.04	1.84	0.65	0.009	0.47	0.90	0.95	0.588	0.79	1.14
**CAD**	1.07	0.729	0.74	1.53	0.96	0.859	0.64	1.44	1.25	0.058	0.99	1.56
**Arterial hypertension**	0.53	0.000	0.40	0.69	0.78	0.091	0.59	1.04	0.60	0.000	0.50	0.71
**Previous MI within 4 months**	xxx	xxx	xxx	xxx	xxx	xxx	xxx	xxx	0.83	0.730	0.29	2.40
**Previous MI within 1 year**	0.39	0.371	0.05	3.09	0.61	0.637	0.08	4.88	1.92	0.160	0.77	4.76
**Previous MI after 1 year**	1.33	0.481	0.60	2.97	0.59	0.407	0.17	2.06	1.63	0.082	0.94	2.81
**Previous CABG**	0.94	0.862	0.46	1.92	0.80	0.625	0.32	1.99	0.86	0.587	0.50	1.49
**Previous cardiac surgery**	1.43	0.166	0.86	2.37	0.48	0.015	0.27	0.87	2.23	0.000	1.61	3.10
**Peripheral vascular disease**	0.94	0.848	0.48	1.82	0.82	0.563	0.42	1.60	1.09	0.710	0.70	1.70
**Carotid disease**	1.07	0.870	0.49	2.32	1.77	0.068	0.96	3.28	1.03	0.905	0.62	1.72
**COPD**	1.06	0.810	0.67	1.67	0.47	0.010	0.26	0.83	1.25	0.126	0.94	1.67
**Pulmonary hypertension**	1.53	0.045	1.01	2.33	0.48	0.007	0.28	0.81	1.58	0.001	1.20	2.08
**Renal disease, GFR <15ml/min**	2.77	0.002	1.44	5.31	0.73	0.508	0.29	1.84	5.11	0.000	3.06	8.53
**Renal disease, GFR <30ml/min**	1.34	0.444	0.64	2.82	0.18	0.003	0.06	0.55	2.26	0.001	1.38	3.71
**Atrial fibrillation**	1.03	0.844	0.78	1.35	1.44	0.013	1.08	1.91	1.49	0.000	1.26	1.77
**Diabetes mellitus**	2.18	0.000	1.58	3.00	1.64	0.009	1.13	2.37	1.86	0.000	1.49	2.32
**Emergency**	3.06	0.000	2.20	4.25	1.58	0.010	1.12	2.24	2.47	0.000	1.96	3.10

	Delirium				Mechanical ventilation >48h			
	OR	p	95% CI		OR	p	95% CI	
**SAVR**	1				1			
**TA-TAVR**	0.37	0.039	0.14	0.95	0.14	0.002	0.04	0.49
**TF-TAVR BE**	0.19	0.000	0.11	0.32	0.09	0.000	0.05	0.17
**TF-TAVR SE**	0.16	0.000	0.10	0.25	0.06	0.000	0.03	0.11
**Year**	1.04	0.449	0.94	1.16	0.92	0.114	0.83	1.02
**Women**	0.99	0.934	0.81	1.21	1.21	0.057	0.99	1.48
**Age**	1.03	0.000	1.02	1.04	1.01	0.079	1.00	1.02
**Logistic EuroSCORE**	1.01	0.460	0.99	1.03	1.00	0.626	0.99	1.02
**NYHA II**	0.98	0.877	0.75	1.28	0.70	0.013	0.52	0.93
**NYHA III or IV**	1.30	0.011	1.06	1.58	1.31	0.006	1.08	1.60
**CAD**	1.19	0.133	0.95	1.51	1.13	0.310	0.89	1.45
**Arterial hypertension**	0.87	0.168	0.72	1.06	0.71	0.000	0.59	0.86
**Previous MI within 4 months**	0.33	0.152	0.07	1.49	0.31	0.132	0.07	1.42
**Previous MI within 1 year**	0.85	0.762	0.29	2.48	1.53	0.400	0.57	4.15
**Previous MI after 1 year**	0.94	0.840	0.51	1.73	1.13	0.686	0.61	2.10
**Previous CABG**	0.95	0.853	0.56	1.62	0.86	0.600	0.48	1.52
**Previous cardiac surgery**	0.99	0.955	0.67	1.45	1.71	0.003	1.20	2.44
**Peripheral vascular disease**	1.29	0.273	0.82	2.04	1.27	0.304	0.81	1.99
**Carotid disease**	1.88	0.009	1.17	3.01	1.30	0.308	0.78	2.17
**COPD**	1.08	0.642	0.79	1.47	1.25	0.155	0.92	1.69
**Pulmonary hypertension**	0.95	0.757	0.69	1.30	1.17	0.323	0.86	1.58
**Renal disease, GFR <15ml/min**	1.52	0.172	0.83	2.78	2.58	0.001	1.51	4.42
**Renal disease, GFR <30ml/min**	1.48	0.134	0.89	2.49	1.16	0.596	0.67	2.00
**Atrial fibrillation**	1.74	0.000	1.45	2.09	1.91	0.000	1.60	2.30
**Diabetes mellitus**	1.55	0.000	1.23	1.96	1.84	0.000	1.46	2.31
**Emergency**	1.54	0.001	1.19	1.99	2.70	0.000	2.13	3.42

	Length of stay				Reimbursement			
	Coeff	p	95% CI		Coeff	p	95% CI	
**SAVR**								
**TA-TAVR**	-4.75	0.000	-7.05	-2.46	2708.97	0.038	152.25	5265.69
**TF-TAVR BE**	-6.88	0.000	-9.06	-4.69	-799.40	0.486	-3049.58	1450.79
**TF-TAVR SE**	-7.22	0.000	-8.95	-5.49	-1666.56	0.047	-3307.30	-25.82
**Year**	-0.63	0.026	-1.19	-0.08	-2387.16	0.000	-3012.63	-1761.69
**Women**	0.17	0.643	-0.55	0.90	331.44	0.542	-734.17	1397.04
**Age**	0.00	0.822	-0.04	0.03	60.76	0.031	5.55	115.96
**Logistic EuroSCORE**	0.00	0.956	-0.11	0.12	-93.42	0.184	-231.34	44.49
**NYHA II**	-0.98	0.131	-2.24	0.29	-1532.03	0.086	-3281.45	217.39
**NYHA III or IV**	0.99	0.081	-0.12	2.11	712.76	0.286	-597.23	2022.75
**CAD**	0.81	0.137	-0.26	1.89	799.58	0.237	-524.62	2123.77
**Arterial hypertension**	-1.00	0.050	-2.00	0.00	-1972.18	0.001	-3163.37	-780.98
**Previous MI within 4 months**	-0.08	0.976	-5.20	5.04	-4074.28	0.109	-9061.51	912.94
**Previous MI within 1 year**	6.23	0.092	-1.01	13.47	6542.95	0.159	-2553.57	15639.47
**Previous MI after 1 year**	1.22	0.415	-1.71	4.14	-315.16	0.843	-3443.09	2812.77
**Previous CABG**	-1.06	0.303	-3.07	0.96	-1410.66	0.271	-3922.59	1101.27
**Previous cardiac surgery**	3.41	0.001	1.33	5.48	5335.14	0.000	2897.25	7773.03
**Peripheral vascular disease**	-0.58	0.552	-2.51	1.34	-1000.96	0.441	-3548.27	1546.35
**Carotid disease**	-0.37	0.694	-2.21	1.47	1350.93	0.332	-1381.23	4083.08
**COPD**	3.18	0.002	1.18	5.17	3930.35	0.011	888.74	6971.96
**Pulmonary hypertension**	2.80	0.035	0.19	5.41	3436.64	0.034	259.47	6613.81
**Renal disease, GFR <15ml/min**	11.18	0.002	4.15	18.20	17013.33	0.002	6163.53	27863.13
**Renal disease, GFR <30ml/min**	4.30	0.003	1.42	7.19	3849.14	0.059	-151.98	7850.27
**Atrial fibrillation**	3.01	0.000	2.07	3.94	3452.52	0.000	2290.50	4614.53
**Diabetes mellitus**	2.45	0.002	0.90	4.00	4084.78	0.000	2005.80	6163.76
**Emergency**	6.66	0.000	5.09	8.24	7720.58	0.000	5770.47	9670.69

	Mortality: Standardized rate	95% CI		Stroke: Standardized rate	95% CI		Major bleeding: Standardized rate	95% CI	
**SAVR**	6.58%	5.51%	7.64%	6.40%	5.32%	7.47%	24.24%	22.03%	26.45%
**TA-TAVR**	4.39%	-0.52%	9.29%	xx	xx	xx	4.66%	0.11%	9.21%
**TF-TAVR BE**	3.57%	1.68%	5.45%	1.31%	0.29%	2.33%	1.81%	0.64%	2.99%
**TF-TAVR SE**	1.52%	0.57%	2.47%	1.33%	0.42%	2.25%	1.29%	0.47%	2.12%

	Delirium: Standardized rate	95% CI		Mechanical ventilation >48h: Standardized rate	95% CI	
**SAVR**	18.20%	15.83%	20.57%	19.72%	17.51%	21.93%
**TA-TAVR**	8.35%	1.72%	14.99%	4.01%	-0.51%	8.54%
**TF-TAVR BE**	4.65%	2.46%	6.84%	2.72%	1.19%	4.25%
**TF-TAVR SE**	4.00%	2.35%	5.65%	1.83%	0.78%	2.87%

	Length of stay: Standardized mean	95% CI		Reimbursement: Standardized mean	95% CI	
**SAVR**	18.94	17.99	19.90	25841.58	24850.03	26833.14
**TA-TAVR**	14.19	11.92	16.45	28550.55	26309.01	30792.10
**TF-TAVR BE**	12.06	10.14	13.99	25042.19	23157.85	26926.53
**TF-TAVR SE**	11.72	10.17	13.27	24175.02	22964.89	25385.16

p-values based on Student's t-test (age, EuroSCORE) or chi-square test.

xx: Values of stroke in TA-TAVR could not be calculated due to a stroke rate of 0.00% in TA-TAVR.

xxx: The Research Data Center of the Federal Bureau of Statistics censored all values that could allow conclusions to be drawn about a single patient or a specific hospital.

BE: balloon-expandable; CABG: coronary artery bypass graft; CAD: coronary artery disease; COPD: chronic obstructive pulmonary disease; EuroSCORE: European System for Cardiac Operative Risk Evaluation; GFR: glomerular filtration rate; MI: myocardial infarction; NYHA: New York Heart Association; SAVR: surgical aortic valve replacement; SD: standard deviation; SE: self-expanding; TA: transapical; TAVR: transcatheter aortic valve replacement; TF: transfemoral.
